# The Swedish bipolar collection (SWEBIC)

**DOI:** 10.1186/s40345-025-00389-4

**Published:** 2025-06-06

**Authors:** Mikael Landén, Erik Joas, Alina Karanti, Lydia Melchior, Olof Zachrisson, Robert Sigström, Elin Hörbeck, Andreas Göteson, Erik Pålsson, Lina Jonsson

**Affiliations:** 1 Department of Psychiatry and Neurochemistry, Institute of Neuroscience and Physiology, Sahlgrenska Academy at University of Gothenburg, Blå Stråket 15, 413 45 Gothenburg, Sweden; 2 Department of Medical Epidemiology and Biostatistics, Karolinska Institutet, Stockholm, Sweden

**Keywords:** Bipolar disorder, Cohort study, Registry, Genetic association study

## Abstract

**Background:**

The Swedish Bipolar Collection (SWEBIC) was launched to investigate the genetic basis of bipolar disorder. Here, we provide a detailed overview of the procedures and assessment tools used during the SWEBIC data collection.

**Methods:**

The SWEBIC collection occurred in two waves, the first from 2009 to 2013, followed by the second wave from 2017 to 2022. Recruitment primarily relied on the Swedish National Quality Register for Bipolar Disorders (BipoläR). Additional sources included the Hospital Discharge Register, an online questionnaire, and identification of individuals with bipolar disorder from other cohort studies. We assessed the diagnostic validity of the BipoläR entries by reviewing randomly selected medical records from the study participants.

**Results:**

Across the two waves, SWEBIC recruited 8580 individuals diagnosed with bipolar disorder, 89 percent from BipoläR. The bipolar disorder diagnoses in BipoläR showed high agreement with medical records (positive predictive value of 0.90). The response rate in BipoläR was higher during the first (61%) than the second wave (23%). Further, the proportion of individuals with subtype 1 was higher in the first wave. Including individuals from other cohort studies, the total number of DNA samples from individuals with bipolar disorder in SWEBIC exceeds 10,000.

**Conclusions:**

Using quality registries to identify patients for large cohort studies facilitates genetic research with high recruitment efficiency and throughput combined with rich phenotypic data. The extensive data and biological samples collected in SWEBIC will continue to be a valuable resource for future studies, advancing our understanding of the genetic basis of bipolar disorder.

**Supplementary Information:**

The online version contains supplementary material available at 10.1186/s40345-025-00389-4.

## Introduction

Bipolar disorder is characterised by episodes of mania and depression interspersed with periods of remission. The condition is highly heritable (~ 60–85%) (Lichtenstein, et al. 2009; McGuffin, et al. 2003) and first-degree relatives to index cases suffer eight times higher risk of developing bipolar disorder (Song, et al. 2015). However, the genetic basis of bipolar disorder is complex. Identifying its underlying genetic factors requires large sample sizes to achieve sufficient statistical power. To address this challenge, the Swedish Bipolar Collection (SWEBIC) was launched in 2009. The first wave of collection (SWEBIC-I), concluded in 2013, successfully recruited over 5000 individuals with bipolar disorder. This cohort has since contributed to several genetic studies of bipolar disorder conducted within the international framework of the Psychiatric Genomics Consortium (PGC), advancing our understanding of the genetic architecture of bipolar disorder (Mullins, et al. 2021; O’Connell, et al. 2025; Sklar, et al. 2011; Stahl, et al. 2019). It is now believed that small effects from many common genetic variants contribute to the risk of developing bipolar disorder. The most recent study from the PGC identified 298 loci associated with bipolar disorder (O’Connell, et al. 2025). Despite the importance of these large-scale studies, much remains to be understood about the genetics underlying bipolar disorder. For example, subphenotypes of bipolar disorder may differ in their underlying biology (Jonsson et al. 2024a, b; Song, et al. 2024; Kalman et al. 2021; Montejo et al. 2025). Genetic factors may also influence treatment response and side effects (Hou, et al. 2016; Joas, et al. 2023; Song, et al. 2016). To gain deeper insights, even larger cohorts with detailed phenotyping are needed. We therefore conducted a second wave of collection, SWEBIC-II, recruiting an additional 5000 individuals with bipolar disorder, which concluded in 2022.

Here, we describe the recruitment and methods used for ascertaining a total of over 10,000 bipolar disorder cases in SWEBIC-I and SWEBIC-II.

## Material and methods

### SWEBIC-I recruitment

From 2009 to 2013, the first wave of the Swedish bipolar collection (SWEBIC-I, https://ki.se/meb/swebic) recruited participants from two primary sources: (1) the Swedish National Quality Register for bipolar disorder (BipoläR) (Pålsson, et al. 2022), and (2) the Swedish Hospital Discharge Register (HDR). An invitation letter was sent to all individuals in the identified patient groups, followed up by a phone call attempt. Those who agreed to participate were scheduled for a telephone interview and provided a blood sample at their nearest lab. In addition, SWEBIC-I included cases from the St. Göran Bipolar Project (N = 272), a longitudinal clinical cohort of individuals with bipolar disorders (Landén, et al. 2025).

#### The Swedish national quality register BipoläR

For a review of Swedish Quality Registers, see Emilsson, et al. (2015). The Quality Register for bipolar disorders—BipoläR—was launched in 2004 with the aim of improving the quality of bipolar disorder care (Pålsson, et al. 2022). Individuals with bipolar disorder treated at psychiatric outpatient clinics in Sweden are eligible for inclusion and annual follow-up in BipoläR. Patients are informed about their registration and have the option to opt-out. It is voluntary for participating outpatient units and treating psychiatrists to contribute data to BipoläR. Data, including diagnoses, registered in BipoläR are entered by the treating psychiatrist, who is usually specialised in the diagnosis and treatment of bipolar disorder and has access to all clinical data. Individuals can be included in BipoläR at any time after their initial bipolar disorder diagnosis. Following initial registration, annual follow-ups are conducted to collect clinical data from the previous 12 months, including occupational status, comorbidities, substance misuse, recurrence of mood episodes, suicide attempts, treatments for bipolar disorder, severity of illness rated with the Clinical Global Impression (CGI-S), health-related quality of life rated with EQ-5D, as well as measurements of lithium serum levels, kidney function, HbA1c, blood pressure, weight and height.

#### Swedish hospital discharge register (HDR)

The HDR is part of the Swedish National Patient Register, maintained by the National Board of Health and Welfare. HDR is based on mandatory reporting of diagnoses made during inpatient health care contacts in Sweden (Ludvigsson, et al. 2011). The HDR includes hospital discharge diagnoses in accordance with the World Health Organization’s International Classification of Diseases (ICD). Since 1973, the HDR has full coverage of inpatient psychiatric care and diagnoses according to ICD-8 (1973–1986), ICD-9 (1987–1996), or ICD-10 (1997–). We identified individuals with bipolar disorder in HDR using a validated algorithm for register-based identification of bipolar disorder, which has a positive predictive value of 0.92 (Sellgren, et al. 2011). This algorithm requires at least two separate discharge diagnoses of bipolar disorder and allows for a maximum of one lifetime schizophrenia diagnosis to exclude individuals with schizophrenia as their main diagnosis.

### SWEBIC-II recruitment

The second wave of recruitment, SWEBIC-II, began in 2017 and concluded in 2022. Bipolar disorder cases were identified and recruited from BipoläR using the same procedure as in SWEBIC-I. We also recruited cases from the general population through an online questionnaire described below. In addition, we used HDR diagnoses to identify individuals with bipolar disorder in other cohort studies not described here: First, from our own PREFECT (Predictors for ECT) study (N = 896) (Clements, et al. 2021; Sigström, et al. 2022, 2020) and the St. Göran Bipolar Project (N = 396) (Landén, et al. 2025). Second, from cohort studies conducted by colleagues, including Karma (a breast cancer study, N = 93) (Gabrielson, et al. 2017), the Swedish Twin Registry (N = 260) (Zagai, et al. 2019), the Stockholm 3 study (a prostate cancer study, N = 74) (Gronberg, et al. 2015), and the LifeGene study (N = 200) (Almqvist, et al. 2011).

#### Online recruitment

In SWEBIC-II, we additionally used a dedicated web portal and a secure online registration form to recruit individuals with bipolar disorder from the general population. To raise awareness of the web portal, we reached out to patient organizations and used other websites and social media platforms. Participants identified themselves using ‘BankID’, a Swedish service for secure online electronic identification and signature. Instead of being interviewed on the phone by a research nurse, participants completed an online questionnaire containing the same questions as the telephone interview. After submission, they were instructed to provide a blood sample at their nearest hospital or laboratory. We validated the diagnosis in subjects who registered online based on national register data as described in the supplementary methods and results.

### Telephone interview

#### Description

Individuals with bipolar disorder recruited from BipoläR and HDR completed a structured telephone interview conducted by trained research nurses (see supplement for data dictionary). Since not all information in BipoläR is available in HDR, the interviews were tailored based on mode of ascertainment. For instance, the interview with participants recruited from HDR included additional questions to determine the subtype of bipolar disorder, which cannot be reliably extracted from ICD-10 codes available in the HDR.

The interview collected information about (1) the study participants’ place of birth, as well as the birthplaces of their parents and grandparents, (2) selected somatic diseases as well as height and weight, (3) nicotine, alcohol, and illicit drug use, and (4) disease course and outcomes, history of psychotic symptoms, comorbid psychiatric conditions, pharmacological treatment responses and side effects, as well as family history of psychiatric disorders.

In SWEBIC-II, we added questions regarding response lithium treatment response (detailed below), questions regarding response and side effects from electroconvulsive treatment, and the Montgomery-Åsberg Depression Rating Scale (MADRS) to the telephone interview.

#### Treatment response and adverse effects

Participants who had used a mood stabilizer (lithium, valproate, lamotrigine, and carbamazepine) were asked to evaluate its effectiveness. The question posed was: “What do you think about its effect? Do not consider any potential side effects.” Based on participant’s responses, the research nurses categorized the answers into one of three predefined response options: (1) Complete treatment response. Mainly free from episodes during treatment. Essentially healthy. “I recovered, it helped me.”; (2) Clearly improved, but suffered continued mood episodes, or needed temporary/continuous additional treatment; and (3) No, or very uncertain treatment effect. From 2018 onwards in SWEBIC-II, the *Retrospective Criteria of Long-Term Treatment Response in Research Subjects with Bipolar Disorder* (Duffy, et al. 2007; Garnham, et al. 2007; Manchia, et al. 2013), also known as the *Alda scale,* was added to the questionnaire for participants who had used lithium for at least 6 months. The Alda scale is designed to measure lithium treatment response specifically in individuals on lithium monotherapy. The scale was modified for individuals treated with a combination of lithium and lamotrigine so that they were first asked if they previously had been on lithium monotherapy. If they had, the Alda scale was conducted for the period during which they were on lithium monotherapy. If they had not, we conducted the Alda scale but deducted two points on question B5 to account for the combination therapy.

There is a risk for treatment emergent switch to mania when antidepressants are used in bipolar disorder (Viktorin, et al. 2014). Therefore, participants were asked if they had experienced a switch to (hypo-)mania within 12 weeks of starting an antidepressant treatment. As weight gain is a common side effect of antipsychotics, participants were also asked whether they had experienced significant weight gain—defined as an increase of at least 7%—within 3 months of starting atypical antipsychotic treatment. Response options included ‘Yes’ or ‘No, or less than 7%’.

#### Data completeness and missingness

Overall, the level of missing data in the structured telephone interviews was low, as the interviews were conducted by trained research nurses using standardized protocols with predefined response options. However, some variability in completeness exists across recruitment waves. For example, variables such as lithium treatment response rated by the Alda scale were introduced during SWEBIC-II and are missing for participants recruited in SWEBIC-I. Moreover, participants recruited from external cohorts (e.g., Karma, STR, Sthlm3, LifeGene) did not complete the telephone interview and therefore lack corresponding interview-based phenotypic data. Annual follow-up data in BipoläR are collected as part of routine clinical care, not specifically for this study, and their completion is not mandatory for either clinicians or patients. Consequently, the completeness of these data may vary across individuals and over time.

### Bipolar disorder subtypes

We used information from the quality registry BipoläR to identify bipolar disorder subtypes. For individuals without BipoläR data, we determined the subtype based on responses from the telephone interview and the online questionnaire. For subjects recruited from PREFECT, we used BipoläR data when available. For subjects recruited from the Swedish Twin Register, the Stockholm 3 study, and Karma, no direct information on bipolar disorder subtype was available. In these cohorts, subtypes were classified based on ICD-10 diagnoses as follows: bipolar disorder subtype 1 (F30.1, F30.2, F31.1, F31.2, F31.6, and F31.7), bipolar disorder subtype 2 (F31.8), and bipolar disorder not otherwise specified (NOS, F31.9).

### Blood sampling and genotyping

Kits for blood sampling were sent to participants, who were asked to have their blood drawn at their nearest hospital or laboratory. Blood samples were collected in EDTA tubes and sent via overnight mail to the Karolinska Institutet Biobank. Upon arrival, the samples were centrifuged for 10 min at 2000 g. Plasma was then apportioned into 225 µl aliquots and stored at − 80 °C. DNA was extracted from whole blood and subsequently stored at − 20 °C.

DNA samples were subsequently sent to the Broad Institute (Massachusetts, US) for genotyping. Individuals in SWEBIC-I ascertained from BipoläR were genotyped using Affymetrix 6.0 (Affymetrix, Santa Clara, CA, USA), Illumina’s OmniExpress and Infinium PsychArray-24 v1.2 BeadChip (Illumina, San Diego, CA, USA), while individuals ascertained from HDR were genotyped using PsychArray. In SWEBIC-II, genotyping was carried out using Illumina Infinium Global Screening Array (GSA). SWEBIC has also been whole exome sequenced (Illumina, San Diego, CA, USA) in collaboration with the Bipolar Sequencing Consortium.

### Diagnostic validity of diagnoses in BipoläR

As most individuals included in SWEBIC were ascertained from the quality register BipoläR, we analysed the validity of the registered bipolar disorder diagnoses in BipoläR.

#### Population for validation study

To select a representative sample for validation of bipolar disorder diagnoses, every 50 th participant consecutively ascertained from BipoläR in SWEBIC-I was selected for validation, up to a total sample of 150 patients. We requested the medical records of these patients from the psychiatric clinics where they had received their care.

#### Clinical assessment

Three board certified psychiatrists (A.K., O.Z., and L.M.) independently reviewed the medical records. The raters were blinded to the diagnoses in BipoläR. The raters first assessed whether patients met criteria for any bipolar spectrum disorder. Second, patients were classified according to the bipolar subtypes: type 1, 2, NOS, cyclothymia, or schizoaffective disorder. If the medical records did not provide sufficient information to determine a specific subtype diagnosis, the raters could select from broader categories coded as “subtype 1 or schizoaffective disorder”, “subtype 1 or 2”, “subtype 2 or NOS” or “other combinations”.

After each rater had classified patients individually, cases were reviewed at consensus meetings attended by the three raters and an additional blinded board-certified psychiatrist (M.L.). In instances where the three raters did not concur, discrepancies were discussed by the group and medical records were re-evaluated. Finally, a consensus diagnosis was reached.

#### Statistical analyses

The validity of diagnoses in BipoläR and the inter-rater agreement for the consensus diagnoses were examined as follows. Diagnostic validity was assessed using positive predictive value (PPV). The board consensus diagnosis agreed upon by our three raters was regarded as the “true positive” value. The diagnoses in BipoläR included both “true” and “false” diagnostic values. We then calculated the proportion of individuals where the diagnosis in BipoläR matched the rater’s consensus diagnoses.

In the first PPV calculations, we included all individuals and the broader diagnosis categories described above were counted as a ‘hit’. In the second PPV calculations, we included only individuals with a non-ambiguous consensus diagnosis. We also assessed the overall reliability and agreement between the consensus diagnoses and the diagnoses recorded in BipoläR using Kappa measurements. Finally, we used Fleiss’ kappa for more than two raters to test the inter-rater reliability for the diagnoses across the board.

Clopper-Pearson exact confidence intervals and *P*-values were calculated using the R packages *PropCI* and *irr* (the kappam.fleiss function), respectively.

### Comparison of participants and non-participants

To evaluate potential differences between SWEBIC participants and the broader BipoläR cohort, we compared the groups with respect to sex, bipolar disorder subtype, medication use, employment status, and global assessment of functioning (GAF) scores using logistic regression (Table S1).

### Ethical considerations

The SWEBIC study was approved by the Regional Ethical Review Board in Stockholm, Sweden (DNR:2008/2009-31/2 and 2016/1363-32). All participants provided written informed consent in accordance with the Declaration of Helsinki.

## Results

### SWEBIC recruitment

**Fig. 1:**
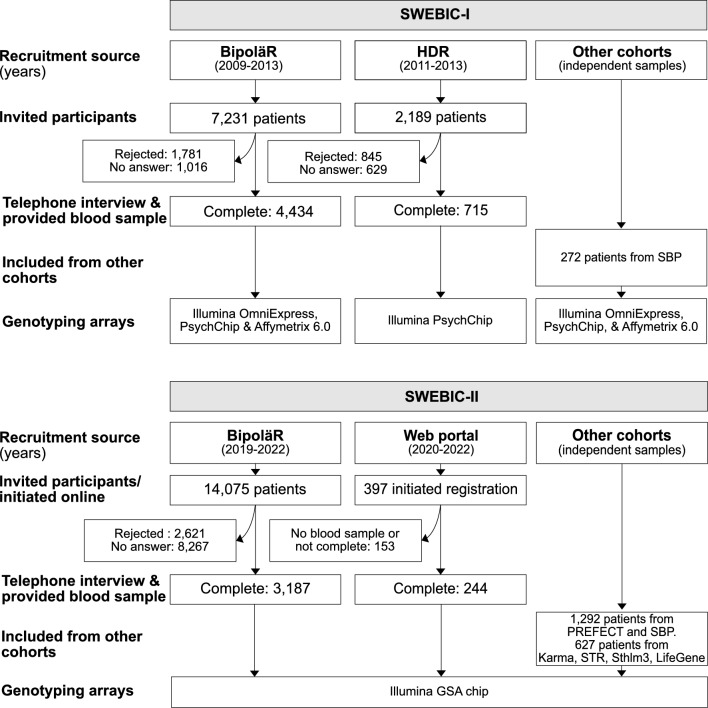
Overview of ascertainment in SWEBIC-I and II

A flowchart of the inclusion and data collection is presented in Fig. 1. Demographics for each mode of ascertainment in SWEBIC-I (BipoläR and HDR) and SWEBIC-II (BipoläR and web portal) are provided in Table 1.

**Table 1:** General description of the SWEBIC samples with genetic and interview information

		SWEBIC-I			SWEBIC-II		
		Ascertainment source			Ascertainment source		
		All	BipoläR	HDR	All	BipoläR	Web portal
		(N = 5149)	(N = 4434)	(N = 715)	(N = 3431)	(N = 3187)	(N = 244)
Description							
Sex	Males N (%)	1917 (37%)	1647 (37%)	270 (38%)	1137 (33%)	1098 (34%)	39 (16%)
Mean year of birth (SD)		1959 (15)	1960 (15)	1958 (14)	1970 (15)	1970 (15)	1980 (13)
Mean age at inclusion (SD)		51.2 (14.5)	50.8 (14.5)	53.4 (14.3)	49.7 (14.9)	50.4 (14.9)	40.9 (12.7)
Genotyping array (Illumina)		Illumina OmniExpress, PsychArray & Affymetrix 6.0	Illumina OmniExpress, PsychArray & Affymetrix 6.0	PsychArray	Global Screening Array	Global Screening Array	Global Screening Array
BD subtype^a^, N (%)	BD-I	2339 (46%)	1894 (43%)	445 (62%)	934 (27%)	881 (28%)	53 (22%)
	BD-II	1682 (33%)	1583 (36%)	99 (14%)	1760 (51%)	1590 (50%)	170 (70%)
	NOS	927 (18%)	816 (18%)	111 (16%)	688 (20%)	667 (21%)	21 (9%)
	Schizoaffective disorder	73 (1%)	73 (2%)	0	46 (1%)	46 (1%)	0
	other/missing	127 (3%)	68 (2%)	60 (8%)	3 (< 1%)	3 (< 1%)	0

^a^Bipolar disorder subtype was determined by the first recorded subtype in BipoläR for subjects ascertained from BipoläR, and by interview data in combination with information from BipoläR (if available) in subjects ascertained from HDR

*BD-I/II/NOS* bipolar subtype type 1/2/Not Otherwise Specified. *HDR* Hospital Discharge Register, *BipoläR* = Swedish National Quality Register for Bipolar Disorder

#### SWEBIC-I

Here, we describe the study participants ascertained from BipoläR and HDR. Invitation letters were sent to 7231 individuals with bipolar disorder in BipoläR, of which 61% (N = 4434) completed the study (Fig. 1). Of the 2797 non-participants in SWEBIC-I, 14% did not reply, and 25% either declined or failed to provide a blood sample. For the HDR-based recruitment, an invitation to participate was sent to 2189 individuals with bipolar disorder, of which 715 subjects (33%) completed the study. Of the 1474 non-participants, 29% did not reply to the invitation and 39% did not want to participate.

The SWEBIC-I collection from BipoläR and HDR yielded a total of 5149 blood samples from individuals with bipolar disorder. Most participants were women (63%), and the mean age at inclusion was 51 years (range 18–81 years). The distribution of bipolar disorder subtypes was 46% for type 1, 33% for type 2, 18% for NOS, and 1% for schizoaffective disorder. As shown in Table 1, ascertainment from HDR included a higher percent of individuals with bipolar disorder type 1 than participants recruited from BipoläR.

#### SWEBIC-II

In total, 5350 individuals were included for genotyping in SWEBIC-II. Here, we describe the recruitment of 3431 (64%) individuals from BipoläR and the online web portal. Of the 14,075 individuals in BipoläR who were invited to participate, 23% (N = 3187) completed the study, 59% did not reply to the invitation, and 19% individuals did not want to participate or did not provide a blood sample. Of the 397 individuals who initiated a registration through the online web portal, 71% (N = 244) completed the questionnaire and provided a blood sample. Out of these 244 subjects, six (2%) did not have a bipolar disorder diagnosis registered in BipoläR (up to 2019) or the National patient register, including inpatient or outpatient diagnoses (up to 2024), as described in the supplementary results (Table S2).

In SWEBIC-II, the mean age at inclusion was 50 years and 67% were women. The distribution of bipolar disorder subtypes was 27% for type 1, 51% for type 2, 20% for NOS, and 1% for schizoaffective disorder. Participants enrolling through the web portal were younger than those ascertained from BipoläR.

### Diagnostic validity in BipoläR

Out of the 150 individuals selected for the BipoläR diagnostic validity study, we successfully retrieved medical records from 111 patients, from which we were able to determine bipolar disorder diagnostic status in 102 individuals (Table 2). A consensus on overarching bipolar diagnosis was reached for 95 of these individuals. Among these, consensus on bipolar subtype was reached for 70 individuals. One individual had missing diagnostic information on bipolar disorder subtype in BipoläR. Table 3 displays the PPV of diagnostic groups for both those with more and less diagnostic information. The PPV’s range from ca 0.8 (type 1) to around 0.1 (NOS). In Table 4, we show the reliability statistics when using BipoläR and the consensus diagnoses as two different ratings. The kappa values are around 0.4–0.5 for all diagnoses except BD-NOS where it is 0. Table 5 show the inter-rater reliability of different diagnostic groups. As there was almost no individual who were deemed not to have a psychiatric diagnosis or a bipolar diagnosis, the variability was extremely low, which is reflected in the low Kappa values. Therefore, we also provide statistics for the raters’ agreement, which demonstrate the substantial agreement between raters. There was over 95% agreement between raters regarding whether the individuals had an affective disorder, and 83% agreement whether individuals had bipolar disorder or not. Agreement for specific bipolar disorder subtype diagnosis was 61% with a Kappa value of 0.6.

**Table 2:** Descriptives of included diagnoses in the diagnostic validity test

	N_tot_ = 111
Consensus BD diagnosis	N (%)
Yes	95 (85.6%)
No	7 (6.3%)
Insufficient data in medical records	9 (8.1%)
Consensus BD subtype	
BD-I	46 (48.4%)
BD-II	15 (15.8%)
BD-NOS	7 (7.4%)
BD-I or BD-II	4 (4.2%)
BD I or BD-NOS; other combination	3 (3.2%)
BD-I or schizoaffective	3 (3.2%)
BD-II or BD-NOS	5 (5.3%)
Schizoaffective	2 (2.1%)
BD diagnoses but insufficient data to determine subtype	10 (10.5%)
BD subtype in BipoläR	
BD-I	45 (40.9%)
BD-II	43 (39.1%)
BD-NOS	19 (17.3%)
Schizoaffective disorder	3 (2.7%)
Missing subtype diagnosis	1

*BD-I/II/NOS* Bipolar disorder subtype 1/2/Not Otherwise Specified

**Table 3:** Positive predictive values of diagnostic groups

	PPV (95% CI)	N
Diagnoses in BipoläR, including the broader categories*		
Any BD spectrum diagnosis	0.93 (0.86–0.97)	102
BD-I	0.85 (0.71–0.94)	81
BD-II	0.64 (0.43–0.82)	84
BD-NOS	0.14 (0.02–0.43)	84
Diagnoses in BipoläR, including only cases with sufficient information to determine subtypes*		
BD-I	0.87 (0.72–0.97)	69
BD-II	0.58 (0.33–0.81)	69
BD-NOS	0.10 (0.00–0.46)	69

*If the information in medical records was insufficient to determine the bipolar disorder subtype, consensus ratings allowed for classification into broader categories (see Methods 2.6.1 and Table 2)

*PPV* Positive Predictive Value, *BD-I/II/NOS* Bipolar disorder subtype 1/2/Not Otherwise Specified

**Table 4:** The reliability statistics when using BipoläR and the consensus diagnoses as two different ratings

Diagnoses	Fleiss Kappa	z	P-value	N
Overall	0.41	4.71	0	69
BD-I	0.49	4.04	0	NA
BD-II	0.53	4.42	0	NA
BD-NOS	− 0.01	− 0.05	0.96	NA
Schizoaffective disorder	0.48	4.03	0	NA

*BD-I/II/NOS* Bipolar disorder subtype 1/2/Not Otherwise Specified

**Table 5:** Inter-rater reliability of diagnostic groups

Diagnostic groups	N	Agreement (%)	Fleiss kappa
The patient has a psychiatric disorder	109	99.1	0
The patient has an affective disorder	108	97.2	0
The patient has a bipolar disorder diagnosis	109	82.6	0.5
- If yes, which bipolar disorder subtype diagnosis	90	61.1	0.6
Psychiatric comorbidity is present	97	67	0.6
Presence of manic episode(s)	100	85	0.8
Presence of hypomanic episode(s)	57	52.6	0.5
Presence of depressive episode(s)	72	77.8	0.2

### Comparison of participants and non-participants

Compared to non-participants in BipoläR, individuals who participated in SWEBIC had a slightly higher psychosocial functioning (GAF scores), were somewhat older, showed a higher prevalence of BD1 and lower prevalence of BD NOS, were more likely to be treated with lithium and other mood stabilizers, and were less likely to receive antipsychotic treatment. However, the magnitude of these differences was small (Table S1).

## Discussion

The SWEBIC study has collected over 10,000 individuals with bipolar disorders for genetic studies. The majority of cases in SWEBIC were ascertained using the Swedish Quality Register for bipolar disorders (BipoläR), which includes a representative sample of the Swedish bipolar disorder population and contains more detailed phenotyping than other national registers (Pålsson, et al. 2022). The comprehensive phenotyping available in BipoläR, including subtypes and disease outcomes, enables analyses of key subphenotypes. Moreover, since treating psychiatrists provide annual reports for each individual, we can incorporate longitudinal data, including information collected after recruitment.

The first wave of the SWEBIC cohort collection was completed in 2013. Using data from SWEBIC-I, we have identified genetic variants associating with lithium treatment response (Song, et al. 2016), studied pharmacogenetic effects on antidepressant treatments (Joas, et al. 2023), explored serum biomarkers of prospective suicide attempts (Sandberg, et al. 2022), investigated the role of copy number variations in bipolar disorder (Bergen, et al. 2012), and examined polygenic scores associating with bipolar disorder subphenotypes (Jonsson et al. 2024a, b; Song, et al. 2024). Further, we recently found that polygenic scores for psychiatric disorders associate with year of the first bipolar diagnosis between the 1970’s and 2016 (Jonsson et al. 2024a, b). These secular changes in the polygenic profile of individuals diagnosed with bipolar disorder is important when interpretating results from genetic studies.

The SWEBIC-I cohort has been included in the genome wide association studies (GWAS) from the PGC-bipolar disorder work group (Mullins, et al. 2021; O’Connell, et al. 2025; Sklar, et al. 2011; Stahl, et al. 2019). Further, a subset of patients from both SWEBIC-I and II has been included in exome sequencing studies in the Bipolar Sequencing Consortium (Jia, et al. 2021; Palmer, et al. 2022), which for instance identified *AKAP11* as a risk gene shared with schizophrenia. Additionally, the SWEBIC-I cohort has been included in other international consortia investigating genetic determinants of brain imaging and proteomics, as well as broader psychiatric genetics (Folkersen, et al. 2020; Kalman, et al. 2021; Ruderfer, et al. 2018; Thompson, et al. 2014; Writing Committee for the Attention-Deficit/Hyperactivity, et al. 2021).

However, larger cohorts are needed to conduct genetic analyses of subphenotypes of bipolar disorder, which is why we launched the second wave of the SWEBIC collection in 2019.

### Ascertainment from the quality register BipoläR

The quality register BipoläR served as the primary recruitment resource for both SWEBIC waves, contributing in total 7621 individuals with bipolar disorder. Despite using the same recruitment strategy, we note significant differences between the first and second collection waves. The response rate was significantly higher during the first wave (60%) compared to the second wave (23%). We experienced increasing challenges in reaching study participants with our approach. While only 14% of the invited individuals in SWEBIC-I did not respond, this figure rose to over 50% in SWEBIC-II. Moreover, a higher number of individuals had no registered telephone number, and those who did were less likely to respond to follow-up calls in the second wave. The lower participation rate may be attributable to changes in communication habits and technology use over time indicating that we may need to adapt recruitment approaches to current trends. Another notable difference across the waves was the distribution of bipolar subtypes: while bipolar disorder type 1 was most common in SWEBIC-I, type 2 became predominant in SWEBIC-II. This trend is consistent with the 2019 annual report from the quality register BipoläR, which shows an increase in subtype 2 diagnoses recorded between 2009 and 2019 (Melchior and Landén 2019).

### Diagnostic validity in BipoläR

Diagnostic validity is crucial in large genetic studies that often include various sources for diagnosis, such as population biobanks, electronic health records, or questionnaires. While psychiatrists across Sweden use DSM criteria to report diagnoses to BipoläR, clinical assessments may vary. We therefore tested the validity of the BipoläR diagnoses.

Our diagnostic validation study of 111 of the included patients revealed a high level of agreement between the psychiatrist ratings and the bipolar disorder diagnoses recorded in BipoläR. This confirms that individuals recruited from BipoläR can be reliably considered as having bipolar disorder. However, agreement on specific bipolar disorder subtypes was lower. As expected, the highest positive predictive value was observed for type 1 (0.85), while the lowest was for the NOS category (0.14). This finding aligns with our previous analyses of BipoläR-data, which showed that diagnosis stability over time was highest for type 1, with less stability for type 2 and NOS (Larsson, et al. 2021).

Importantly, the moderate agreement between raters and the bipolar subtype diagnoses registered in BipoläR may not be as concerning as it appears. First, our Kappa values for bipolar disorder type 1 (0.49) and type 2 (0.53) are comparable to those reported in the DSM-5 field trials, where the pooled Kappa value was 0.56 for bipolar disorder type 1 and 0.46 for schizophrenia (Regier, et al. 2013). Second, the sparse information in medical records often limited our raters’ ability to accurately assess subtypes. It is therefore possible that the diagnoses in BipoläR are accurate, but the independent reviewers were unable to verify them due to the limited clinical details available in the records.

### Ascertainment from HDR and web portal

In the first wave, a smaller portion of cases were ascertained from HDR using our validated algorithm (Sellgren, et al. 2011), which captured a higher percentage of bipolar disorder type 1 compared to other sources. This outcome was expected, as the algorithm requires at least two inpatient admissions, which are more common in type 1 than in type 2 or NOS. In the second wave, we introduced a web portal as an alternative recruitment method to boost enrolment. The online recruitment offered the advantage of eliminating the need for telephone interviews with research nurses. However, relatively few individuals registered online. Future cohort collections using a web portal would likely require a more extensive information campaign or targeted advertisement to enhance participation.

### Telephone interview/register linkage

Trained nurses conducted structured telephone interviews with over 8000 individuals recruited through BipoläR and HDR. The questionnaire was designed to capture information not available in BipoläR, improving phenotype harmonization across other cohorts—a key challenge in large-scale genetic analyses of bipolar disorder subphenotypes. Further, Sweden’s extensive national registers can be linked to the SWEBIC cohort, offering valuable opportunities for future research. For example, the Prescribed Drug Register provides detailed data on all dispensed medications, allowing for comprehensive analyses of treatment patterns and medication adherence. Similarly, the HDR captures data on hospital admissions, diagnoses, comorbid conditions, and suicide attempts. By linking these registers with SWEBIC, we can significantly expand the depth and scope of available data, allowing for more robust investigations into the genetic, clinical, and environmental factors influencing bipolar disorder.

### Strengths and limitations

A major strength of SWEBIC is its large sample size of individuals with bipolar disorder, combined with rich phenotypic data. This was made possible by using the BipoläR quality register BipoläR as the primary recruitment source. However, a limitation is that diagnoses in SWEBIC, while based on real-world clinical assessments, were made by treating clinicians rather than following a standardized research protocol. Although our validation of the BipoläR diagnoses showed reasonable agreement between expert ratings and recorded diagnoses, this approach can still introduce variability in diagnostic accuracy and subtype classifications. Another limitation affecting a small subset of participants recruited from external cohorts is the use of ICD-10 codes to define bipolar disorder subtypes in the absence of direct subtype information. In this subgroup, potential misclassification between bipolar I and II may occur due to ambiguity in codes such as F31.6 and F31.7. Another limitation is that SWEBIC primarily includes subjects of European descent, which may limit the generalisability of findings across different populations. While the recent bipolar disorder GWAS by PGC (O’Connell, et al. 2025) has begun addressing cross-ancestry generalisability, future studies need to focus more closely on this issue.

### Conclusion

By using Swedish national registers as recruitment sources, we successfully ascertained a large cohort of individuals with bipolar disorder with rich phenotype data. The results from our studies, along with collaborations within international consortia, have significantly enhanced the understanding of the genetic architecture of bipolar disorder. Looking ahead, SWEBIC will remain a resource for ongoing and future genetic studies aimed at unravelling the genetic underpinnings of bipolar disorder.

## Supplementary Information

Additional file 1

## Data Availability

The Public Access to Information and Secrecy Act in Sweden prohibits us from making individual level data publicly available. Anonymized data can be requested through the bipolar disorder working group of the Psychiatric Genomic Consortium.

## Abbreviations

BipoläR: The Swedish National Quality Register for bipolar disorders
HDR: Hospital Discharge Register
GSA: Global Screening Array
SBP: St. Göran Bipolar Project
PREFECT: Predictors for ECT study
Karma: Karolinska Mammography Project for Risk Prediction of Breast Cancer
STR: The Swedish Twin Registry
Sthlm3: The Stockholm 3 study (a prostate cancer study)
