# Supplementary material for: The Swedish bipolar collection (SWEBIC)
Source: Int J Bipolar Disord. 2025 Jun 6;13:20. doi: 10.1186/s40345-025-00389-4 (PMC12144026; doi:10.1186/s40345-025-00389-4)
Supplement: Supplementary file 1 — Additional file 1 [file 40345_2025_389_MOESM1_ESM.pdf]

SUPPLEMENT

The Swedish Bipolar Collection (SWEBIC)

Mikael Landén, MD, PhD*[1, 2], Erik Joas, PhD[1], Alina Karanti, MD, PhD[1], Lydia Melchior, MD[1], Olof Zachrisson, MD, PhD[1], Robert Sigström, MD, PhD[1], Elin Hörbeck, MD[1], Andreas Göteson, MD[1], Erik Pålsson, PhD[1], Lina Jonsson, PhD[1]

[1] Department of psychiatry and neurochemistry, Institute of Neuroscience and Physiology, Sahlgrenska Academy at University of Gothenburg, Sweden
[2] Department of Medical Epidemiology and Biostatistics, Karolinska Institutet, Stockholm, Sweden

METHODS

Comparison of participants and non-participants

These analyses were conducted to test how representative the group participating in SWEBIC was compared to all subjects included in the national quality register for bipolar disorder (BipoläR). We included all subjects ascertained from BipoläR in SWEBIC-I/II and compared this group with all other subjects with registrations in BipoläR since the start in 2004 up until 2019. The analyses included 7,586 subjects from SWEBIC compared to 16,960 non-SWEBIC subjects in BipoläR. We compared the groups based on the following measures: year of birth, sex, global assessment of functioning (GAF), employment, bipolar disorder subtype, and medications (lithium, mood stabilizers, antipsychotics, and antidepressants). All the included measures were extracted from the yearly reports in BipoläR, where we included the first registered measure for those with multiple registrations.

Web portal diagnosis validation (SWEBIC-II)

In SWEBIC-II, we included a web portal for ascertainment of self-reporting through an online questionnaire. All subjects answered a question if they had been diagnosed with bipolar disorder by a medical doctor. To validate that they indeed had a bipolar diagnosis, we have linked subjects in SWEBIC to the Swedish registers BipoläR, hospital discharge register (HDR), the national outpatient register (OPR), and the Prescribed Drug Register (PDR). We included the diagnoses F-30/31 in ICD-10, and 296* in ICD-8/9 for bipolar disorder diagnoses in HDR and OPR. Based on information in these registers, we regarded a diagnosis

as validated if they had received a bipolar disorder diagnosis in HDR, OPR or were included in BipoläR. We also tested if we could identify additional subjects who had been prescribed lithium (ATC code: N05AN01) in PDR.

RESULTS

Comparison of participants and non-participants

Compared to non-participants, SWEBIC study participants exhibited slightly higher functioning (GAF scores), were somewhat older, had a higher prevalence of BD1 and lower prevalence of BD NOS diagnoses, were more likely to be treated with lithium and other mood stabilizers, and less likely to receive antipsychotic treatment. However, the observed differences were small (Table S1).

Web portal diagnosis validation (SWEBIC-II)

Of the 244 subjects who completed the web portal, for six subjects we did not identify a bipolar disorder diagnosis in HDR, OPR, or that they were included in BipoläR as shown in Table S2. We did also not identify that they had received a prescription of lithium between 2005 and 2024. Thus, in total 6 subjects (2%) lacked a bipolar disorder diagnosis in the web portal. We did not have information about diagnoses in primary care, and cannot exclude the possibility that the subjects may have received a bipolar disorder diagnosis there.

Table S1. Results comparing SWEBIC included and non-included subjects from BipoläR.

	SWEBIC		BipoläR (excl. SWEBIC)			
**Continuous phenotypes**	**N (w info)**	**Mean (SD)**	**N (w info)**	**Mean (SD)**	β	**P-value**
Year of Birth	7,586	1963.6 (15.6)	16,958	1967.7 (17.3)	-0.014	**<0.001**
GAF-F	6,060	67.3 (13.4)	13,279	64.7 (13.5)	0.014	**<0.001**
**Binary phenotypes**		**N (%)**		**N (%)**		
Sex (Women)	7,586	4,843 (63.8)	16,960	10,585 (62.4)	0.061	**0.032**
Employment > 50%	6,843	2,546 (37.2)	15,007	5,340 (35.6)	0.070	**0.021**
Bipolar subtype	7,552		16,873		-0.114	**<0.001**
Type 1		2,761 (36.6)		5,697 (33.8)		
Type 2		3,147 (41.7)		6,894 (40.9)		
NOS		1,520 (20.1)		3,848 (22.8)		
Schizoaffective syndrome		124 (1.6)		434 (2.6)		
Medications						
Lithium	7,572	4,200 (55.5)	16,895	7,876 (46.6)	0.355	**<0.001**
Mood stabilisers	7,506	6,481 (86.3)	16,625	13,453 (80.9)	0.399	**<0.001**
Antipsychotics	7,431	2,787 (37.5)	16,508	6,871 (41.6)	-0.172	**<0.001**
Antidepressants	7,438	3,341 (44.9)	16,514	7,290 (44.1)	0.031	0.265

BipoläR=National quality register for bipolar disorder, GAF-F=Global Assessment of Functioning – Functioning. NOS=not otherwise specified. Bold indicates P-values < 0.05.

Table S2. Descriptives of registered diagnosis for subjects included in SWEBIC-II through the web portal.

		Validated diagnosis				
	**Web portal**	**BipoläR**	**HDR**	**OPR**	**Lithium prescription (PDR)**	**No registered BD diagnosis or lithium prescription**
N	244	87 (36%)	117 (48%)	235 (97%)	176 (73%)	6 (2%)
Mean (SD) number of diagnoses/ prescriptions			4.6 (8)	33.3 (30)	52.7 (60)	

BipoläR=National quality register for bipolar disorder, HDR=Hospital Discharge Register, OPR=National Outpatient Register, PDR=Prescribed Drug Register.

Table S3. Data dictionary for the included variables in SWEBIC.

**REGISTER VARIABLES**		
**Variable**	**Data**	**Source**
Bipolar disorder subtype	Type 1/Type 2/NOS/Schizoaffective disorder manic type	The quality register BipoläR, supplementary interview questions for participants recrutied online or from the National Patient Register
Psychiatric comorbidity	ICD-codes	The National Patient register
Suicide attempts	ICD-codes	The National Patient register
Sick leave	Longitudinal data	The LISA database, Statistics Sweden
Unemployment	Longitudinal data	The LISA database, Statistics Sweden

**TELEHONE INTERVIEW**			
**Variable**	**Question Text**	**Response Options**	**Notes**
**Background**			
Country of birth	Were you born in Sweden?	Yes, No (specify country), Don't know/Don't want to answer	Background
Place of birth	In what town did you and your parents live when you were born?	Open text, Don't know/Don't want to answer	Background
County of birth	Which county were you born in?	Open text, Don't know/Don't want to answer	Background
Hospital of birth	Which hospital were you born in?	Open text, Don't know/Don't want to answer	Background
Other birth location	Were you born somewhere else (e.g., at home)?	Yes, No, Don't know/Don't want to answer	Background
Mother born in Sweden	Was your biological mother born in Sweden?	Yes, No (specify country), Don't know/Don't want to answer	Background
Father born in Sweden	Was your biological father born in Sweden?	Yes, No (specify country), Don't know/Don't want to answer	Background

**Medical history variables**			
Height	How tall are you?	cm/ Don't know / Don't want to answer	
Weight	How much do you weigh today?	kg/ Don't know/ Don't want to answer	
Diabetes diagnosis	Do you have diabetes?	Yes, No, Don't know, Don't want to answer	Requires physician diagnosis
Type of diabetes	If yes, do you know which type of diabetes?	Type 1, Type 2, Don't know, Don't want to answer	Conditional on Diabetes diagnosis = Yes
Hypothyroidism	Do you have hypothyroidism?	Yes, No, Don't know, Don't want to answer	Includes follow-up on whether lithium-induced
Hyperthyroidism	Do you have hyperthyroidism?	Yes, No, Don't know, Don't want to answer	
Migraine	Do you have migraines?	Yes, No, Don't know, Don't want to answer	Includes age of onset
Epilepsy	Do you have epilepsy?	Yes, No, Don't know, Don't want to answer	Includes age of first seizure and treatment
Severe head injury	Have you had a head injury that rendered you unconscious?	Yes, No, Don't know, Don't want to answer	Includes number of times, duration, and cause
Brain infection	Have you ever had an infection to your nervous system?	Yes, No, Don't know, Don't want to answer	Includes age of infection
Stroke	Have you ever had a stroke?	Yes, No, Don't know, Don't want to answer	Includes age of onset
High blood pressure	Do you have high blood pressure?	Yes, No, Don't know, Don't want to answer	Requires treatment
High cholesterol	Do you have high cholesterol level and are treated for this?	Yes, No, Don't know, Don't want to answer	
Heart disease	Do you have heart disease (e.g., infarction, angina)?	Yes, No, Don't know, Don't want to answer	
**Medication**			
Lithium use	Are you currently or previously treated with lithium?	Yes, No, Don't know/Don't want to answer	Includes duration

Past use of lithium	Have you previously been treated with lithium?	Yes (specify duration), No, Don't know/Don't want to answer	Includes reason for discontinuation
Current use of mood stabilizers	Are you currently being treated with mood stabilizers?	Yes (specify), No, Don't know/Don't want to answer	Includes lithium, valproate, lamotrigine, carbamazepine, others
Current antidepressant use	Are you currently being treated with antidepressants?	Yes (specify), No, Don't know/Don't want to answer	Includes drug name
Current antipsychotic use	Are you currently being treated with antipsychotics?	Yes (specify), No, Don't know/Don't want to answer	Includes drug name
Other current medications	Are you currently taking other medications (non-psychiatric)?	Yes (specify), No, Don't know/Don't want to answer	Excludes short-term painkillers
**Treatment response**			
Mood stabiliser response	What do you think about the effect of the treatment? Do not take possible side effects into account.	Complete treatment response. Essentially free from episodes during treatment. Essentially healthy. "I got better, it helped me."**/Clearly improved. But continued episodes or temporary/ongoing additional treatment needed./No or very doubtful treatment effect./Don't know / Don't want to answer.**	Separate response for lithium, valproate, lamotrigine, karbamazepine
The Alda scale			Administered in SWEBIC II if at least 6 months lithium use
**Side effects**			
Antidepressant-induced switch	Have antidepressants caused hypomania/mania?	Yes (specify drug), No, Don't know/Don't want to answer	
Weight gain from antipsychotics	Have you experienced significant weight gain from antipsychotics?	Yes (≥7%), No, Don't know/Don't want to answer	
Hypothyroidism following lithium treatment			See variable 'Hypothyroidism'
**ECT**			

ECT treatment	Have you ever received ECT (electroconvulsive therapy)?	Yes (depression, mania, other), No, Don't know/Don't want to answer	Includes year of first treatment, total number, and effect
Memory after ECT	Memory problems after most recent ECT series?	None, Temporary, Persistent, Don't know/Don't want to answer	Also assesses memory before treatment
Current memory problems	Do you currently have memory problems?	None, Temporary, Persistent, Don't know/Don't want to answer	Includes autobiographical memory loss
Other ECT side effects	Did you experience other side effects from ECT?	Muscle soreness, Confusion, Anxiety upon waking, Other (specify)	
**Psychiatric history**			
Current symptoms	Do you have any current symptoms? (Depressive, manic, etc.)	None, Depressive, Elevated mood, Don't know/Don't want to answer	Multiple responses allowed.
MADRS-S	MADRS-S form	9 items rated 0-6	Administered if 'yes' to currently depressed
Functioning between episodes	How have you felt between episodes or since your last episode?	Fully recovered, Partially recovered, Not recovered, Don't know/Don't want to answer	
Age at first psychiatric symptoms	How old were you when you first had psychological problems?	Numeric, Don't know/Don't want to answer	Followed by detailed questions on type and context of psychotic symptoms
Panic attacks	Have you ever had a panic attack?	Yes, No, Don't know/Don't want to answer	Includes follow-up on onset, avoidance, and impact
Psychotic episodes	Have you ever lost touch with reality (been psychotic)?	Yes, No, Don't know/Don't want to answer	Follow-up on episode type and interviewer assessment
**Suicidality**			
Suicide attempts	Have you ever attempted suicide?	Yes (1-2 times), Yes (3 or more), No, Don't know/Don't want to answer	
Suicidal ideation	Have you ever felt that suicide was your only option?	Normal zest for life, Life feels meaningless, Thoughts of suicide, Plan to commit suicide	MADRS-S item
**Family history**			

Family history of psychiatric illness	Does anyone in your biological family have psychiatric illness?	Yes (specify), No, Unknown, Adopted	Includes detailed family mapping
**Substance use**			
Current smoking status	Do you currently smoke?	Yes, Occasionally, No, Don't know/Don't want to answer	Includes amount and history
Current snus use	Do you currently use snus?	Yes, Occasionally, No, Don't know/Don't want to answer	Includes amount and history
Alcohol use frequency	How often do you drink alcohol?	Almost every day, Weekly, Monthly, Yearly, Never, Don’t know, Don’t want to answer	Also asks about peak drinking period
Alcohol-related problems	Has alcohol ever caused you problems?	Yes, No, Don't know/Don't want to answer	
Tried to stop drinking	Have you tried to stop drinking but failed?	Yes, No, Don't know/Don't want to answer	
Lifetime drug use	Have you ever used narcotic drugs?	Yes, No, Don't know/Don't want to answer	Includes type and frequency
Recent drug use	Have you used narcotic drugs in the past 12 months?	Yes, No, Don't know/Don't want to answer	Includes type
Ever used narcotics	Have you ever used narcotics?	Yes, No, Don’t know, Don’t want to answer	Includes type and frequency
